# Isolation and identification of *Legionella *spp. from different aquatic sources in south-west of Iran by molecular &culture methods

**Published:** 2016-12

**Authors:** Mohammad Tabatabaei, Zahra Hemati, Maryam-o-sadat Moezzi, Negar Azimzadeh

**Affiliations:** Department of Pathobiology, School of Veterinary Medicine, Shiraz University, Shiraz, Iran

**Keywords:** *Legionella pneumophila*, Legionnaires’ diseases, manmade water sources

## Abstract

*Legionella pneumophila*, the causative agent of Legionnaires’ diseases (LD) is usually transmitted to humans via inhalation of aerosols from contaminated natural and manmade water sources. These organisms may become fatal especially in immunocompromised patients and LD is the one of the important disease from a public health perspective. This survey investigated the frequency of *Legionella *spp. including *L. pneumophila, *in some cold and warm water systems in South-West of Iran by culture and PCR methods. Thirty four water samples were collected from diverse water supply systems. After acid and heat treatments of samples, inoculated onto buffered charcoal yeast extract agar. Isolated colonies were confirmed by morphological and biochemical tests. Then the isolates were examined for *icmO*, *sidA *and *lidA *genes by PCR assay. This study showed that frequency of *L. pneumophila *was 4 by culture and 14 by PCR. PCR method to be efficient and sensitive test for rapid detection of these organisms in environmental water sources. This study emphasizes the need for effective infection control and prevention strategies to minimize the risk from exposure to potential pathogens such as *Legionella *spp. and to create a safe working environment.

## INTRODUCTION

*Legionellae *spp. are facultative intracellular Gram negative, catalase and oxidase negative bacteria that may cause acute respiratory infection in humans such asLegionnaires’ disease (LD) and Pontiac fever which is a mild flu-like lung illness[1]. Aquatic environments are the most important source for *Legionella *infections [2]. Human infections are acquired by the inhalation of aerosols produced from natural and artificial aquatic environments, such as cooling tower systems (CTS), water distribution devices, potable hot water systems (HWS), fishponds and equipment of respiratory treatments [3]. It is important from a public health perspective to survey industrial manmade water systems for the presence of *Legionella *[4]. In recent years, the occurrence rate of LD has been reported to be on the increase in many countries [5].

In current, over 90% of LD cases are caused by infection of *L. pneumophila *[1]. LD is also recognized as a major form of travel associated pneumonia, these cases present a major problem because of difficulties in identifying the main source of infection [6]. *L. pneumophila *often protected from harsh environmental conditions within biofilms or intracellular growth amoebae and the biofilms have a major impact on manmade water systems, making the understanding of the bacterial physiological adaptation in biofilms a fundamental step towards their annihilation [7]. There is some limitation on the selection of an appropriate drug for treatment of infections caused by *Legionella *spp [8]. The increased presence of the large-scale, manmade aquatic reservoirs has possible led to the increased human's exposure to *Legionella *and subsequently an increased incidence of *Legionella *infection in the last half of the 20th century [9]. There are numerous reports of colonization of water system in large buildings such as hospitals and fish pools and the current knowledge about the epidemiology of LD is based mainly on data gathered from studies of outbreaks [6]. Water sampling for *Legionella *is an essential component of investigations of LD outbreaks and sampling is useful in identifying potentially contaminated water system with these bacteria used to identify the sources of the implicated etiologic agent [10]. Persistence of *L. pneumophila *in water systems is a public health risk and reflects in the greater research focus on this bacterium [9]. The standard method of detection of *Legionella *spp. in environmental sample is culture on Buffered Charcoal Yeast Extract (BCYE) agar supplemented with L-cysteine [11, 12]. In addition, the polymerase chain reaction (PCR)-based method can be particularly useful for analysis of environmental water samples and provides a platform capable of rapid screening of samples for trace levels of *legionella *spp [1]. In this study, PCR methods were designed for various genetic loci including *Legionella*-specific virulence determinants (*icmO*, *sidA *and *lidA*) genes [13]. Despite efforts to keep water systems free of *Legionella *spp., these pathogens are still causing infection throughout the world wide, including, Iran [3, 14- 16]. Therefore we decided to study some of manmade water sources in view of the presence of *Legionella *spp. including *L. pneumophila, *by two methods of culture and PCR. Although, this study was conducted to survey the prevalence of *Legionella *contamination of various manmade water systems and a larger study and reports from other parts of the country may help in determining the true significance of LD in Iran.

## MATERIALS AND METHODS

**Water sample collection procedures: **During a one year period starting in September 2013, a total of 34 water samples were collected from diverse water supply systems using sterile Erlenmeyer Flask which had been sterilized at 180ºC for 2 hours. Samples were collected from distinct sites at nine CTS (26/47%) from (four different hospitals, three educational departments, two shopping centers) and 17 HWS (50%) from (four different hospitals, seven different universities, three different abattoirs, one shopping center, one dormitory, one library) and eight different fish pools (23/53%) in Shiraz, Iran, as study points. In the manmade water systems, urban drinking water source.were used. Sample temperature was measured at the time of sampling. All of these samples were transferred to the laboratory of microbiology department of pathobiology, faculty of veterinary medicine, Shiraz University in less than two hour and were kept in the refrigerator (4ºC) until the time of processing.

**Sample Concentrations &Standard Culture Method: **To detect *Legionella *spp.,

2.5 liters of the water samples were concentrated by filtration separately through a 0.22 μm pore-size polycarbonate filter (Advantec Tokyo Co. Ltd., Tokyo, Japan), in sterile condition in a biological safety cabinet and the membranes were resuspended in 5 mL of distilled water. After centrifugation in 2500 rpm for 15 min, the supernatant was subjected to both acid and heat treatment for selective inhibition of non-*Legionella *bacteria. From all concentrated and treated samples, aliquots of 0.1 ml were plated onto BCYE Agar medium (Difco Laboratories, Detroit, Mich., USA) supplemented with L. cysteine and the plates were incubated in candle jar (3-5% CO2) at 37ºC in a humidified atmosphere and examined for 4–14 days for the presence of *Legionella *spp. colonies. After incubation, colonies showing characteristics of *Legionella *species such as grayish-white, shiny colonies were selected. Gram stain was done to show the thin, faintly stained filamentous Gram-negative morphology. Suspected colonies were subcultured on BCYE agar with and without L-cysteine and non-selective media, such as sheep-blood agar and MacConky agar for verification. Isolates which grew on BCYE agar with L. cysteine, but not on the other media, were considered supposed *Legionella*. Subsequently the identification of *Legionella *spp. were done by biochemical tests. Strains unable to grow on media without L-cysteine were further identified by PCR analysis of part of the *icmO*, *sidA *and *lidA *genes [13].

**DNA extraction & PCR assay: **To extract the DNA, freshly grown bacterial colonies from solid media (BCYE agar plates) were suspended in 100 μl of deionized sterile water in an ependorf tube, gently vortexed and frozen in liquid nitrogen and heated in boiling water three times. The DNA was further extracted and purified using Promega DNA Extraction Kit (Promega Wizard® Genomic DNA Purification Kit, Madison, USA) according to manufacturer’s instructions. The DNA concentration was determined by measuring the optical density at 260 nm. PCR was performed to investigate *Legionella *genes using different primers. Master mixture was prepared for a 25 μl of reaction consisting of 2.5 μL of 10× PCR buffer, 0.2 μM of each primer, 0.2 mM of each dNTPs,

1 units of *Taq *DNA polymerase (Fermentas) and 2 μL of DNA samples. For amplification, an initial denaturation step for 5 min at 95°C followed by 33 cycles of 95ºC for 30s, 52ºC for 1 min and 72ºC for 90s., and a final extension at 72°C for 5 min in a thermocyler (Techne Co. UK). PCR-amplified DNA fragments were visualized by electrophoretic separation of 8 μL PCR products in a 1.5% agarose gel containing 0.5 ug ethidium bromide per milliliter of gel. Gels were viewed on a UV transilluminator (UV Tech, France), and DNA fragment sizes were compared with a 100-bp DNA ladder (Pharmacia Biotech Co., Ltd., Tokyo, Japan).

Oligonucleotide primers were designed from the partial sequences of the *icmO*, *sidA *and *lidA *genes of *L. pneumophila *[13]. In this study, DNA of *L. pneumophila*, NCTC 12821 (FEPTU, HPA Center for Infections, London, UK, kindly donated from Dr. Nikaeen Associated professor, Isfahan University of Medical Sciences) was used as positive control and molecular grade water without DNA as the negative control. Sequences and sizes of primers are shown in Table 1.

**Table 1:** Oligonucleotide primers used in this study

**Gene**	**Primer sequence (5′-3′)**	**Product size(bp)**	**Reference**
*lidA*	TCACATCAAGTTAAAACATCAG ATGCTCACGCTGTAAGGATTG	882	
*icmO*	AATTTTCGGTTCAACGGGTAG CAGTGCGGGTAATAAAACCAC	763	Gilmour et al (2007)
*sidA*	GAC GAA CCA ACG GTC AGG AT TGC CGC CAG TAC CAA AGA CA	330	

**Statistical analysis: **All statistical analysis were made with SPSS software. Results were analyzed by McNemar test and one way analysis of variance.

## RESULTS

This survey presents the results of an environmental investigation on *Legionella *spp. that was carried out on the numerous water samples taken from fish pools, drain of CTS and HWS within the building of department of eight hospitals, seven universities, eight pools, three abattoirs, three shopping centers, three educational departments, one dormitory and one library in Shiraz, Iran. Water samples included sediments and biofilms in these infrastructures. Inoculating samples on BCYE and nonselective media resulted in isolation of several strains of filamentous gram negative bacteria. These gram-negative strains were identified based on morphology of the colonies and growth on the Blood agar and MacConky agar and biochemical tests. The results of growth on blood and MacConky agar, biochemical tests, such as Catalase, Oxidase, Urease, Gelatin, Motility, and gram staining were −, −, +, +, −, and −, respectively. Isolates were positive by oxidase, hippurate hydrolysis, gelatin tests and had weaker reaction with catalase were suspected to be *L. pneumophila. *The culture method revealed 7.3% (4 of 34) of the water samples among the manmade water systems studied being positive for *L. pneumophila*. Nine strains of *Legionella-like *organisms which were isolated by culture from water sources studied, four strains were later identified by biochemical tests as *L. pneumophila*. The results of this study also showed only one strain (2.9%) to be isolated from 9 CTS, two strains (5.9 %) to be isolated from 17 HWS and one strain (2.9%) to be isolated from 8 fish pool samples were identified as *L. pneumophila *by culture.

**Figure 1:**
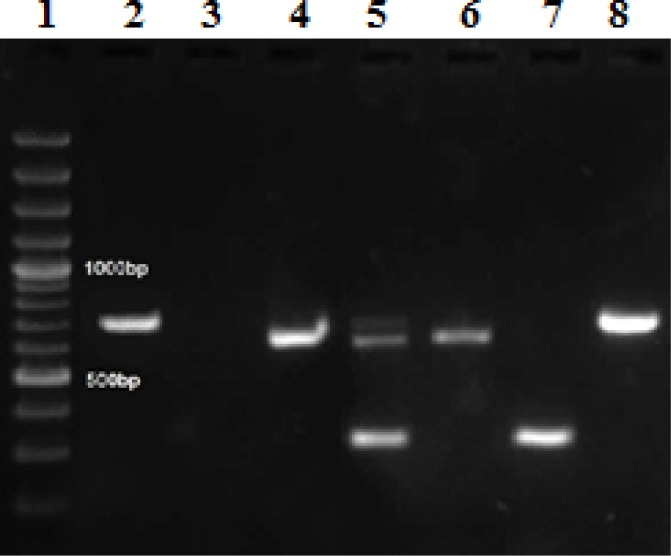
Amplified products of *Legionella *L. pneumophila. by PCR. Lane 1, 100 bp DNA ladder; Lanes 2 and 8, positive sample of *lidA *gene; Lane 3, negative control; Lanes 4 and 6, positive samples of *icmO *gene; Lane 5, positive control; Lane 7, positive sample of *sidA* gene

Extraction of DNA by snap-chill method (10 minutes boiling and chilling in crushed ice) turned out to be an efficient method of DNA preparation for PCR as the results of this method was equally good and comparable to that of PCR employing pure DNA extracted by DNA extraction kit. PCR-based detection assays for *L. pneumophila *were conducted on DNA extractions of all water samples. The *sidA*, *icmO *and *lidA *genes of *L. pneumophila *were amplified using specific primers and showed amplicons about 330, 763 and 882 bp respectively on agarose gel (Fig. 1). Of the total of 34 samples, 14 (41.18%) were PCR positive for one, two or three of these genes. The results in Table 2 show that the following genes were detected in all samples. About 41.18% of the samples contained *L. pneumophila *that 50% (7 of 14) were isolated from HWS, 14.3% (2 of 14) were isolated from CTS and 35.7% (5 of 14) were found in fish pool waters. The numbers of *L. pneumophila *positive samples from the hospitals, universities, pools, abattoirs, shopping center, dormitory, library and educational departments were 3, 3, 5, 2, 1, 0, 0 and 0, respectively. Seven (20.6%) isolates were positive for *lidA *gene, two (5.9%) isolates were positive for *icmO *gene, one isolate (2.9%) was positive for *lidA *and *sidA *genes, one isolate (2.9%) was positive for *sidA *and *icmO *genes and three isolate (8.8%) were positive for three genes.

From the HWS, 5.9% of the samples had temperatures of 15–30ºC, 23.5% had temperature between 30–45ºC, 58.8% had temperature between 45–60ºC, and 11.8% of them had temperature over 60ºC. *Legionella *spp was detected in 7 of the 17 samples of HWS, 71.4% of these samples had temperatures between 45-60ºC and 26.6% of them had temperatures below 45ºC.

Statistical analyses (McNemartest) indicated there was a significant difference between PCR and culture results of these water sources (P<0.006). The statistical analysis using a one-way ANOVA test also confirms there was statistically significant difference among the temperatures of the samples taken from the HWS, CTS and those taken from the fish pools (P<0.001).

**Table 2:** PCR products of different genes in different bacterial sources

Sources	Hot water system			Cooling tower			Fish pools		Total
	*lidA*	*icmO*	*LidA,sidA,icm*	*LidA,sidA,icmO*	*SidA,icm*	*lidA*	*LidA,sidA*	*icmO*	
Hospitals	0	0	1	1	1	0	0	0	3(21.43%)
Universities	2	0	1	0	0	0	0	0	3 (21.43%)
Fish pools	0	0	0	0	0	3	1	1	5 (35.71%)
Abattoirs	1	1	0	0	0	0	0	0	2 (14.29%)
Shopping centre	1	0	0	0	0	0	0	0	1 (7.14%)
Dormitory	0	0	0	0	0	0	0	0	0
Library	0	0	0	0	0	0	0	0	0
Education Department	0	0	0	0	0	0	0	0	0
Total	4	1	2	1	1	3	1	1	14 (100%)

## DISCUSSION

LD is a common form of nosocomial and community-acquired pneumonia in human, that majority of this infection is caused by *L. pneumophila *[12, 17]. *L. pneumophila *is a dangerous pathogenic bacterium can cause serious lower respiratory tract infection especially in immunocompromised peoples and may become fatal for them [8]. *Legionella *spp. present a significant public health risk when they colonize manmade water systems operating between 20-45ºC and grow to high concentrations [18]. Infection of these bacteria is not contagious, and inhaling the contaminated aerosol is the only way of *Legionella *infections thus having the clean air is the first important parameter to prevent LD [19]. *Legionella *spp. are ubiquitous distributed in different water environments and have been isolated from a wide variety of water samples [3, 17]. These pollution sources can serve as a focus for the spread of *Legionella *spp. For the majority of sporadic LD cases the source of infection remains unknown [12]. Increased standardization of LD reporting, and more precise follow-up of LD events, would help generate stronger, more comparable evidence on LD sources, contributing factors, and control measure effectiveness [21]. The disease may result from exposure to these bacteria in different resources that are not yet considered to be a source of infection [10]. Therefore, some environmental water samples from potentially implicated building water systems were studied to identify the possible source of exposure to pathogenic *Legionella *bacteria. In this study we used a combination of PCR and culture methods for identifying the environmental sources of *Legionella *spp. The PCR assay used in this study to target the *icmO*, *sidA *and *lidA *genes showed to be a rapid and sensitive test. In our study, frequency of positive *L. pneumophila *in 34 examined water samples were 4 (7.3%) by culture, while PCR could detect 14 (41.18%) of these samples. All samples that were found positive with the culture method were also positive with the PCR assay. Ten of 34 water samples (29.4%) tested in their study were negative by culture but positive by PCR. Control reaction included pure DNA sample of reference strain. In a study conducted by Li et al., (2015), *Legionella *spp*. *were detected in 35.7% of collected industrial CTS samples from 22 factories during the 19-month study period, and showed a high degree of pollution of *Legionella *in industrial CTS in China.

Statistical analyses (McNemartest) indicated there was a significant difference between PCR and culture results of these water sources (P<0.006). Several authors showed PCR to have a higher rate of detection than culture-based methods (3, 16, 20). Baghal Asghari et al., (2013) reported the rates of detection of *Legionella *spp. to be 66% (29 of 44) of the water samples by PCR compared to 23% (10 of 44) by culture. Furthermore, prevalence of *Legionella *spp. could be affected by temperature because that lower temperature of the CTS contains lower number of positive cases [14]. *Legionella *spp. present a significant public health risk when they colonize manmade water systems operating between 20-45oC and grow to high concentrations [18]. In our study, the mean temperature of the water samples positive for *Legionella *spp. was higher compared to the mean temperature of the negative samples (44.3°C versus 37.6°C). In addition, the most number of positive water samples were belonged to HWS, number of *Legionella*- positive samples was much lower in CTS. We found that lower temperatures could explain the lower prevalence of *Legionella *spp*. *These findings is agree with research in Greece, where the temperature, which was found not only within the limits favorable to developing *Legionella *spp. (20-45°C) but also for development and proliferation of the bacteria, and other microorganisms as well [6]. In any case, the use of internal control reduced the chances of false negative results.

Our results demonstrated high prevalence of *Legionella *in industrial manmade water systems and we can conclude that the results of our study provide data and some insight in the possible detected *Legionella *spp. in water sources in Shiraz, which would be of value in investigating any further outbreak of LD in the geographical region studied. Thus improving control and prevention strategies of *Legionella*, containing routine monitoring of industrial manmade water systems are urgently needed. For future studies, we wish to conduct prospective surveillance in Shiraz and obtain *L. pneumophila *isolates from patient samples.
